# The Aquaporin 1 Inhibitor Bacopaside II Reduces Endothelial Cell Migration and Tubulogenesis and Induces Apoptosis

**DOI:** 10.3390/ijms19030653

**Published:** 2018-02-26

**Authors:** Helen M. Palethorpe, Yoko Tomita, Eric Smith, Jinxin V. Pei, Amanda R. Townsend, Timothy J. Price, Joanne P. Young, Andrea J. Yool, Jennifer E. Hardingham

**Affiliations:** 1 Molecular Oncology, Basil Hetzel Institute, Queen Elizabeth Hospital, Woodville South, SA 5011, Australia; helen.palethorpe@adelaide.edu.au (H.M.P.); yoko.tomita@sa.gov.au (Y.T.); eric.smith@adelaide.edu.au (E.S.); joanne.young@adelaide.edu.au (J.P.Y.); 2 Adelaide Medical School, University of Adelaide, Adelaide, SA 5005, Australia; jinxin.pei@adelaide.edu.au (J.V.P.); amanda.townsend@sa.gov.au (A.R.T.); timothy.price@sa.gov.au (T.J.P.); andrea.yool@adelaide.edu.au (A.J.Y.); 3 Medical Oncology, Queen Elizabeth Hospital, Woodville South, SA 5011, Australia

**Keywords:** angiogenesis, aquaporin-1 (AQP1), bacopaside II, endothelial cell migration

## Abstract

Expression of aquaporin-1 (AQP1) in endothelial cells is critical for their migration and angiogenesis in cancer. We tested the AQP1 inhibitor, bacopaside II, derived from medicinal plant *Bacopa monnieri*, on endothelial cell migration and tube-formation in vitro using mouse endothelial cell lines (2H11 and 3B11) and human umbilical vein endothelial cells (HUVEC). The effect of bacopaside II on viability, apoptosis, migration and tubulogenesis was assessed by a proliferation assay, annexin-V/propidium iodide flow cytometry, the scratch wound assay and endothelial tube-formation, respectively. Cell viability was reduced significantly for 2H11 at 15 μM (*p* = 0.037), 3B11 at 12.5 μM (*p* = 0.017) and HUVEC at 10 μM (*p* < 0.0001). At 15 μM, the reduced viability was accompanied by an increase in apoptosis of 38%, 50% and 32% for 2H11, 3B11 and HUVEC, respectively. Bacopaside II at ≥10 μM significantly reduced migration of 2H11 (*p* = 0.0002) and 3B11 (*p* = 0.034). HUVECs were most sensitive with a significant reduction at ≥7.5 μM (*p* = 0.037). Tube-formation was reduced with a 15 μM dose for all cell lines and 10 μM for 3B11 (*p* < 0.0001). These results suggest that bacopaside II is a potential anti-angiogenic agent.

## 1. Introduction

Angiogenesis is critical for the continuing growth and progression of solid tumours and is largely driven by tumour production of vascular endothelial growth factor (VEGF) to stimulate endothelial cell proliferation [1]; the process is also critically dependent on the migration of endothelial cells to form the new vascular networks (reviewed in [2]). Cell migration involves rapid changes in cell volume and shape mediated by the transmembrane protein aquaporin-1 (AQP1), which allows water flow across the plasma membrane in response to osmotic gradients created by ion transport and actin de-polymerization. Water (and ion) influx through AQP1 can increase hydrostatic pressure and is proposed to cause local expansion of the plasma membrane, followed by actin re-polymerization to stabilise the cell membrane protrusion [3]. The increased permeability is important in allowing actin remodelling and cytoskeleton rearrangement required for migrating cells [4]. AQP1 is over-expressed in many human cancers including those of breast, colon and prostate and was shown to be a prognostic marker for poorer survival outcome in colon cancer patients (reviewed in [5]). Previously, we reported AQP1 over-expression in a proportion of patients’ colorectal cancer tumours compared to matched normal mucosa, with the highest AQP1 expression in the endothelial cells of the microvessels in colon tumour tissue. This was consistent with higher AQP1 expression in human umbilical vein endothelial cells (HUVECs) compared to colon cancer epithelial cell lines [6]. We have also shown that pharmacological inhibition of AQP1 results in impaired migration, invasion and tubulogenesis in vitro [6]. Evidence is thus accumulating based on our own work and others [5] to suggest that AQP1 is a prime target for anti-cancer therapy.

Extracts from the plant *Bacopa monnieri* have long been used in traditional Hindu medicine for improving memory and treating anxiety and epilepsy [7]. We previously examined a purified extract from this medicinal plant, bacopaside II, and showed using the *Xenopus* oocyte expression system that it specifically blocked AQP1-mediated water transport [8]. We also found that it significantly reduced migration of a colon cancer cell line expressing relatively high levels of AQP1 [8]. 

The anti-angiogenesis drugs bevacizumab and aflibercept, in combination with chemotherapy and regorafenib as monotherapy, have shown improved progression-free survival and overall survival in several phase III trials [9,10,11,12]. However a significant number of patients do not achieve any meaningful benefit from this VEGF-targeted therapy. This could in large part be due to inherent resistance mechanisms that allow cancers to evade the effects of VEGF inhibition, or via induced release of compensatory pro-angiogenic factors (reviewed in [13,14]): alternative types of anti-angiogenic therapies are thus required. This is the first study to investigate the effects of bacopaside II on endothelial cell migration and tubulogenesis and provides evidence that bacopaside II is a potential anti-angiogenic therapy for the treatment of cancer.

## 2. Results

### 2.1. Endothelial Cells Express the Bacopaside II Target AQP1

**Figure 1:**
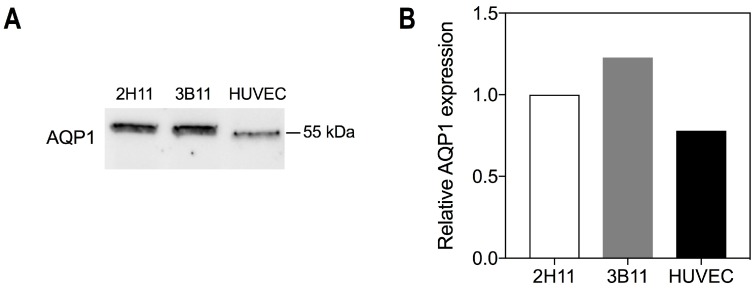
AQP1 expression in endothelial cell lines. (**A**) Western immunoblot confirming AQP1 protein expression in mouse endothelial cell lines 2H11 and 3B11 and human endothelial cell line HUVEC; (**B**) Quantification of AQP1 bands in (**A**), standardized relative to 2H11 and normalised to total protein.

AQP1 was expressed in all three endothelial cell lines, 2H11, 3B11 and HUVEC, as determined by Western immunoblot. The molecular weight was approximately 55 kDa, consistent with post-translational modification (glycosylation) [15] (Figure 1A). Relative levels of AQP1 expression were similar between the cell lines at 1.0, 1.23 and 0.78 for 2H11, 3B11 and HUVEC, respectively (Figure 1B). 

### 2.2. Bacopaside II Reduced Endothelial Cell Viability and Increased Apoptosis

**Figure 2:**
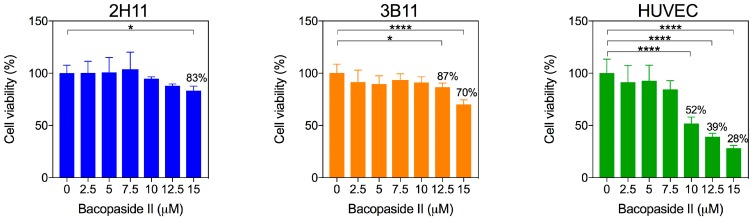
Bacopaside II decreased viability. Cells were treated with bacopaside II for 20 h, and viability was determined by the MTS assay. Data are shown as the mean ± SD (*n* = 6 per treatment group). Asterisks represent significant differences relative to the vehicle control (* *p* < 0.05; **** *p* < 0.0001).

Bacopaside II induced a dose-dependent reduction in cell viability for all three endothelial cell lines (Figure 2). There was a significant decrease in cell viability compared to the vehicle control at 15 μM for 2H11 cells (83% viability, *p* = 0.037), at 12.5 μM (87% viability, *p* = 0.017) and 15 μM (70% viability, *p* < 0.0001) for 3B11 cells and at 10 μM (52% viability), 12.5 μM (39% viability) and 15 μM (28% viability) for HUVECs (*p* < 0.0001).

**Figure 3:**
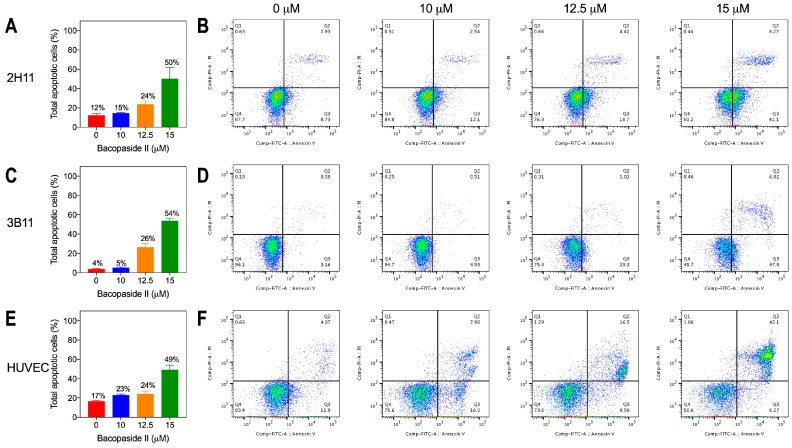
Bacopaside II increased endothelial cell apoptosis as determined by flow cytometry. Histograms (left) show percentages of apoptotic cells (early and late) in a representative experiment for data shown in the scatter plots at the different concentrations of bacopaside II. Results are shown for 2H11 (**A**,**B**), 3B11 (**C**,**D**) and HUVEC (**E**,**F**). In (**A**,**C**,**E**), results shown as the mean ± SD; in (**B**,**D**,**F**), scatter plots show population gates of viable cells (left lower quadrant), or cells either in early apoptosis (right lower quadrant), late apoptosis (right upper quadrant), or necrosis (left upper quadrant).

The proportion of cells undergoing apoptosis following treatment with bacopaside II was measured to determine whether this accounted for the reduction in cell viability at higher concentrations. Apoptosis was determined following 8 h of treatment with bacopaside II to correspond to similar time points monitored in the wound closure assay. There was a dose-dependent increase in the number of cells undergoing apoptosis (early and late) for both mouse endothelial cell lines and HUVECs (Figure 3). The increase in apoptosis with 10, 12.5 and 15 μM bacopaside II relative to that with vehicle was 3%, 12% and 38%, for 2H11 (Figure 3A,B), 1%, 22% and 50% for 3B11 (Figure 3C,D) and 6%, 7% and 32% for HUVEC (Figure 3E,F).

### 2.3. Endothelial Cell Morphology Was Altered by Bacopaside II

**Figure 4:**
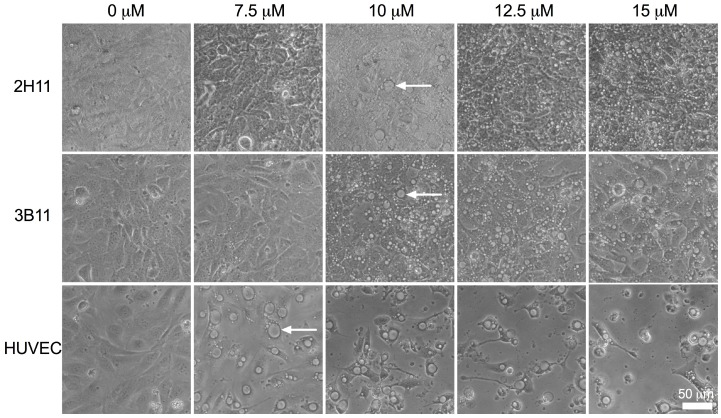
Endothelial cells treated with bacopaside II formed intracellular vacuoles. Endothelial cells following 20 h-treatment with various concentrations of bacopaside II. Vacuoles appeared at 10 μM for 2H11 and 3B11 and at 7.5 μM for HUVECs as indicated by white arrows. (200× magnification, scale bar = 50 μm).

Treatment with bacopaside II induced similar morphological changes in all three endothelial cell lines (Figure 4). Large intracellular vacuoles were evident at concentrations of bacopaside II that induced a decline in cell viability, ≥12.5 μM for 2H11 and 3B11 and ≥10 μM for HUVEC, but also at doses that did not reduce cell viability, 10 μM for 2H11 and 3B11 and 7.5 μM for HUVEC. 

### 2.4. Endothelial Cell Migration Was Reduced by Bacopaside II

**Figure 5:**
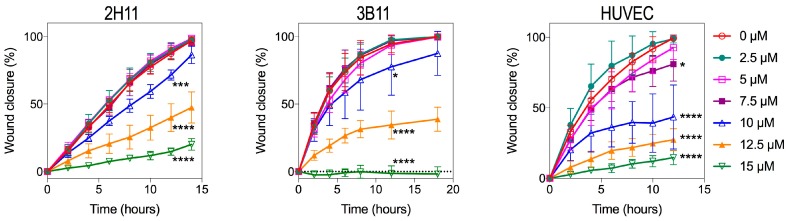
Endothelial cell migration was inhibited by bacopaside II. Wound area for 2H11, 3B11 and HUVEC was measured using NIS-Elements software (Nikon, Tokyo, Japan) and the percentage closure calculated relative to that at Time 0 for each cell line. Data are the mean ± SD of six replicates from a representative experiment. *p*-values are for closure relative to vehicle at 12 h, determined by one-way ANOVA with the Bonferroni test (* *p* < 0.05); *** *p* ≤ 0.0002; **** *p* < 0.0001).

We assessed the effects of various concentrations of bacopaside II on endothelial cell migration in a scratch wound assay. Figure 5 shows a dose-dependent slowing of migration with bacopaside II for all three endothelial cell lines. This was significant for 2H11 at 10, 12.5 and 15 μM with 72% (*p* = 0.0002), 40% (*p* < 0.0001) and 15% (*p* < 0.0001) closure respectively compared to 88% with vehicle. The 3B11 line behaved similarly with closure of 78% (*p* = 0.034), 35% (*p* < 0.0001) and 0% (*p* < 0.0001) at 10, 12.5 and 15 μM compared to 95% with vehicle. The human endothelial cell line HUVEC was most sensitive to the effects of bacopaside II with closure of 81% (*p* = 0.037), 44% (*p* < 0.0001), 27% (*p* < 0.0001) and 15% (*p* < 0.0001) at 7.5, 10, 12.5 and 15 μM as compared to 99% closure with vehicle.

### 2.5. Bacopaside II Inhibited Endothelial Cell Tube Formation

**Figure 6:**
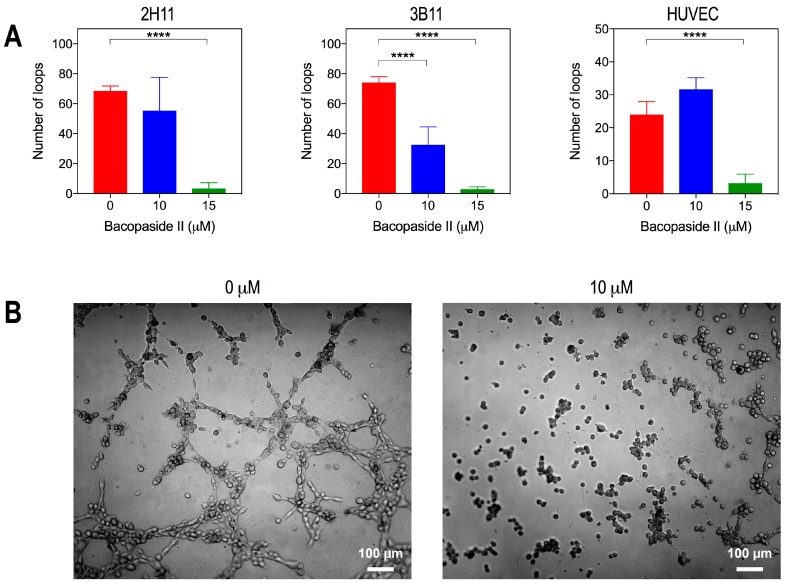
Endothelial cell tube formation was inhibited by bacopaside II. (**A**) 2H11, 3B11 and HUVEC were treated with 0, 10, or 15 μM bacopaside II for 2–3 h (2H11 and 3B11) or 20 h (HUVEC) and the number of loops counted. Data are the mean ± SD of six replicates from two independent experiments. Asterisks represent *p*-values relative to the vehicle control (**** *p* < 0.0001). (**B**) Representative images of 3B11 on matrigel-coated plates treated with 0 and 10 μM bacopaside II.

We used an in vitro tube-formation assay to assess the anti-angiogenic potential of bacopaside II. Tube-forming ability, as measured by the number of loops assembled, was significantly reduced compared to vehicle with 15 μM bacopaside II for 2H11 (vehicle, 69 loops; 15 μM, 3 loops) (Supplementary Data), with both 10 and 15 μM for 3B11 (vehicle, 74 loops; 10 μM, 33 loops; 15 μM, 3 loops) and with 15 μM for HUVECs (vehicle, 24 loops; 15 μM, 3 loops; *p* < 0.0001) (Figure 6A). Representative images for 3B11 after 2–3 h of treatment (Figure 6B) show that cells treated with 10 μM bacopaside II clustered into small aggregates without any capacity to form tubes.

**Figure 7:**
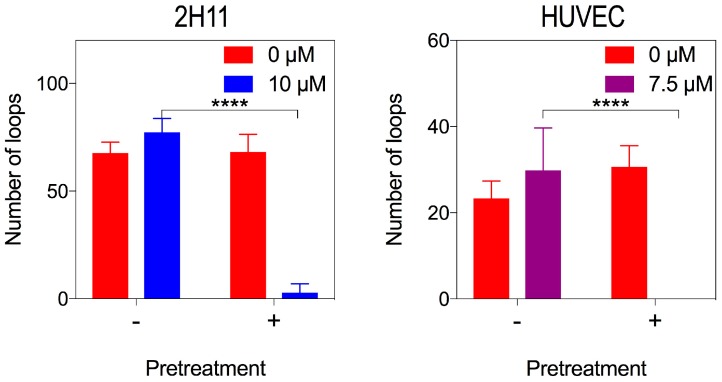
Lower concentrations of bacopaside II inhibited endothelial tube formation of 2H11 and HUVEC after pre-treatment with the drug. 2H11, and HUVEC were cultured in either normal medium or were pre-treated for 20 h with bacopaside II at 0 and 10 μM for 2H11 or 7.5 μM for HUVEC. Untreated and pre-treated cells, resuspended either in vehicle or bacopaside II, were used in the tube formation assay and the number of loops counted. Data are the mean ± SD from two independent experiments (*n* = 6). Asterisks represent *p*-values for bacopaside treated cells without pre-treatment relative to with pre-treatment (**** *p* < 0.0001) (two-way ANOVA with the Bonferroni test).

Since the 15 μM dose reduced viability and increased apoptosis, we further investigated tube formation at lower doses of bacopaside II. Unlike results for 3B11, doses of bacopaside II of 10 μM for 2H11 and 7.5 μM for HUVEC had no effect on tube formation despite significantly slowing migration. We considered that bacopaside II had insufficient time to have its effect on these cells due to the rapidity of the tube formation assay. Since the MTS assay showed that after 20 h of treatment, there was no significant loss of cell viability at these concentrations, we pre-treated 2H11 with 10 μM and HUVEC with 7.5 μM bacopaside II for 20 h and used these cells in tube formation assays with the same dose of bacopaside II. Pre-treated cells failed to form tubes with these doses, while cells that only received the treatment for the duration of the assay did form tubes (Figure 7). This confirmed that bacopaside II could inhibit tube formation independently of inducing apoptosis and needed time to cross the cell membrane to access the intra-cellular binding site of AQP1.

## 3. Discussion

This is the first study to assess the anti-angiogenesis potential of the AQP1 inhibitor bacopaside II. We assessed its effects on cell viability, apoptosis, morphology, migration and tube-forming ability in mouse endothelial cell lines 2H11 and 3B11 and human endothelial cell line HUVEC. In summary, both mouse endothelial cell lines showed increased apoptosis, reduced migration and significant inhibition of tube-formation at 15 μM. At 10 μM, there was no loss of viability and no significant induction of apoptosis, yet migration was inhibited, and for 3B11, tubulogenesis was also inhibited. 2H11 cells required pre-treatment with 10 μM bacopaside II to show inhibition of tubulogenesis. HUVECs were the most sensitive showing reduced viability, increased apoptosis and inhibited migration at 10, 12.5 and 15 μM and inhibition of tubulogenesis at 15 μM. In HUVECs, migration was reduced even at 7.5 μM, with no loss of viability, and following 20 h of pre-treatment, tube formation was also inhibited.

The results of this study suggest that bacopaside II is a potential anti-angiogenesis therapy; it is effective at reducing endothelial cell migration and tube formation at doses below those that induce cell death. This is consistent with our previous work showing that AQP1 activity and HT29 colon cancer cell migration were significantly blocked at concentrations that did not significantly reduce viability over 24 h [6,8]. In addition to the apoptosis-inducing activity of bacopaside II, we found that treatment of 2H11 and HUVEC with a non-cytotoxic dose of bacopaside II inhibited tube formation, provided cells had adequate length of exposure to the agent. The requirement for pre-treatment suggests that there is a latency period, which could be the time for bacopaside II to transit across the cell membrane to access the proposed intracellular binding site on AQP1. In addition, we have found that bacopaside II was predicted to have the most favourable binding energy at a position occluding the cytoplasmic side of the AQP1 water pore; this was not the case for AQP4 [8], and we are currently analysing the binding probabilities of other AQPs. Bacopaside II and other active constituents of *B. monnieri* extract are reported to modulate the activity of P-glycoprotein (P-gp), and this interaction may alter the bio-availability of any P-gp substrate drug, a point to be considered for co-administration in vivo [16].

While anti-angiogenic drugs may inhibit angiogenesis by targeting any of the steps involved in neovascularisation including degradation of extracellular matrix, migration, proliferation or tube formation, many act by inducing vascular endothelial cell apoptosis as demonstrated both in vitro and in vivo [17,18,19,20]. Others have shown that certain anti-angiogenic inhibitors can induce autophagy [19,20]. Here, we show that blocking AQP1 with bacopaside II induces apoptosis in a dose-dependent manner and provide the first evidence that morphological changes induced by bacopaside II are consistent with the appearance of autophagic vacuoles. Currently-used anti-angiogenic drugs, such as bevacizumab, target the vascular endothelial growth factor (VEGF) signalling pathway and have become a mainstay in the treatment of malignant disease. However, despite a number of randomised clinical trials showing a clinical benefit of anti-VEGF drugs in combination with chemotherapy [21,22], many patients do not respond, and others become resistant to such therapy [23]. Furthermore, VEGF inhibition may lead to a more invasive phenotype with an increased number of metastases [24]. Since aquaporins play roles in transcellular fluid transport and cell migration, it is reasonable to consider whether inhibiting AQP1 would be expected to have problematic side-effects. However, in the very rare human cases where there is an inherited bi-allelic inactivating mutation in AQP1, the only clinical consequence is an impaired renal concentrating ability, which would be a concern only in conditions of dehydration [25,26]. Thus, AQP1 presents as a plausible therapeutic target, which could be acted upon to decrease potential for metastasis while not contributing to widespread effects in other tissues. The current study provides a strong foundation for future in vivo investigations that will evaluate whether bacopaside II could be used clinically as an anti-angiogenic therapy for cancer patients.

## 4. Materials and Methods

### 4.1. Cell Lines and Cell Culture

The mouse endothelial cell lines 2H11 and 3B11 were purchased from the American Type Culture Collection (ATCC, Manassas, VA, USA) and maintained in complete medium of DMEM with 10% foetal calf serum (FCS) (Equitech-Bio, Inc., Kerrville, TX, USA), 1% penicillin-streptomycin (Life Technologies, Grand Island, NY, USA) and 1% glutamax (Life Technologies). Human umbilical vein endothelial cells (HUVEC) (PromoCell, Heidelberg, Germany) were cultured in endothelial growth medium (In vitro Technologies, Noble Park North, VIC, Australia) according to the supplied protocol.

### 4.2. Analysis of AQP1 Expression by Western Immunoblot

Whole-cell lysates were prepared, and 20 μg of protein were resolved by denaturing electrophoresis on 12% Mini- Protean TGX precast polyacrylamide gels (Bio-Rad Laboratories, Hercules, CA, USA), transferred by the Trans-Blot Turbo Transfer System (Bio-Rad) and immune-stained using 1:1000 rabbit anti-human AQP1 polyclonal IgG (ab168387, Abcam, Cambridge, UK). Immunoreactivity was detected using the appropriate horseradish peroxidase-conjugated IgG and visualized using enhanced chemiluminescence (Bio-Rad). Bands were quantified relative to total protein loaded using Image Lab software (Bio-Rad).

### 4.3. MTS Viability Assay

Endothelial cells were seeded in complete medium (2H11 and 3B11) or endothelial growth medium (HUVEC) at 1 × 10^4^ cells per well of a 96-well plate and were incubated overnight. Cells were treated with various concentrations of bacopaside II for 20 h. The cell viability was determined using the CellTiter 96^®^ AQueous Non-Radioactive Cell Proliferation Assay (Promega, Madison, WI, USA) according to the manufacturer’s instructions and absorbance read at 492 nm. Results were calculated as the mean absorbance normalized to the vehicle control.

### 4.4. Apoptosis Assay

Cells (1 × 10^5^) were seeded in six-well plates and left for 48 h to reach confluence. Apoptosis controls were treated with paclitaxel (400 nM) for 24 h prior to the Time 0 collection. Necrosis controls were prepared by heating harvested cells to 63 °C for 30 min. Cells were treated either with medium (untreated), vehicle (1% methanol) or 10, 12.5 or 15 µM bacopaside II. Cells were collected at 0 and 6 h after treatment, stained with the Annexin-V-FLUOS staining kit (Roche Diagnostics, Mannheim, Germany) as per the manufacturer’s instructions and run on the BD FACSCanto II cell analyser (BD Biosciences, San Jose, CA, USA). Gating with cell doublet exclusion and analysis was performed using FlowJo software v 10.4.

### 4.5. Scratch Wound (Migration) Assay

Cells were seeded according to growth rate at either 1 × 10^4^ per well for 2H11, 4 × 10^4^ for 3B11 or 6 × 10^4^ per well for HUVEC in 96-well plates in their respective medium for 6 h and then serum starved overnight in medium with just 2% FCS and including 1 μg/mL mitomycin C (Sigma, St. Louis, MO, USA) (the mix referred to herein as “mitomedium”), to inhibit cell proliferation. The following day, a circular wound was made in the cell monolayer with a p10 pipette tip. Loose cells were removed and medium replaced with fresh mitomedium for 1 h followed by replacement with treatment, either vehicle (1% methanol) or various concentrations of bacopaside II in mitomedium. Cells were monitored every 2 h with the area of wound closure determined at each time point.

### 4.6. Endothelial Tube Formation (Angiogenesis) Assay

Mouse endothelial cells or HUVECs were seeded onto a thin layer (10 μL) of matrigel in a 96-well angiogenesis μ-plate (Ibidi, Martinsried, Germany) at 1.5 × 10^4^ cells per well either in vehicle (1% methanol) or bacopaside II at 10 or 15 μM made in complete medium (2H11 and 3B11) or endothelial growth medium (HUVEC). The numbers of loops formed were counted at peak tube formation, 2–3 h for mouse endothelial cells and 20 h for HUVECs. For pre-treatment experiments, cells were either cultured in normal medium, or pre-treated with vehicle or bacopaside II at lower concentrations, 10 μM for 2H11 and 7.5 μM for HUVEC, followed by harvesting of cells, resuspension in vehicle or bacopaside II and seeding on matrigel, with loops counted at peak formation time as before.

### 4.7. Statistical Analysis

The numerical data are presented as the means ± standard deviation. Cell viability, wound closure and tube formation assays were analysed by ANOVA with the Bonferroni post-hoc test.

## 5. Conclusions

The results show that bacopaside II significantly inhibits migration and angiogenesis of mouse and human endothelial cells and induces apoptosis. These actions of bacopaside II may be mediated by the blocking of aquaporin 1 activity and require further investigation both in vitro and in vivo.

## Supplementary Materials

Supplementary materials can be found at http://www.mdpi.com/1422-0067/19/3/653/s1.

## Abbreviations

AQP1	Aquaporin 1
MTS	(3-4,5-Dimethylthiazol-2-yl)-5(3-carboxymethoxyphenyl)-2-(4-sulfophenyl)-2*H*-tetrazolium inner salt
HUVEC	Human umbilical vein endothelial cell
ATCC	American Type Culture Collection
FCS	Foetal calf serum
VEGF	Vascular endothelial growth factor
